# Impact of COVID-19 Confinement on the Health-Related Habits of People at High Risk of Type 2 Diabetes

**DOI:** 10.3390/nu15040841

**Published:** 2023-02-07

**Authors:** Darío Ochoa Esteban, Carmen Martin-Ridaura, Carmen Berlinches-Zapero, Dolores Ruiz-Fernández, Vanessa Sanz-Martín, Rosario Gavira-Izquierdo, Aitana Muñoz-Haba, Sebastià March, Mercedes Ceinos-Arcones

**Affiliations:** 1 Madrid Salud, Madrid City Council, 28007 Madrid, Spain; 2 Cooperativa APLICA, 28014 Madrid, Spain

**Keywords:** diabetes type 2, prediabetes, health promotion, general lockdown, COVID-19, lifestyle, diet, physical activity, obesity, Mediterranean diet, eating habits

## Abstract

The general lockdown decreed in Spain due to the COVID-19 pandemic interrupted the ALAS health promotion intervention aimed at the population at high risk of suffering from type 2 diabetes. We conducted a descriptive study in 2020 through a telephone survey and a comparison with baseline data to determine the impact of confinement on the lifestyles of the participants. We collected sociodemographic variables and conducted assessments before/after confinement on general health status and lifestyle (sleep, physical activity and diet). Additionally, weight, BMI and adherence to a Mediterranean diet were assessed. Descriptive statistical analyses, comparisons of pre–post confinement data and logistic regression were carried out. A total of 387 individuals responded. Among them, 31.8% reported a worse perception of health after confinement, and 63,1% reported no change. Regarding exercise, 61.1% reduced their weekly physical activity time. Regarding diet, 34,4% perceived worse quality, and 53.4% reported no change, despite the fact that 89.4% declared changes in their eating practices. Weight and BMI decreased by 3,1%, and adherence to the Mediterranean diet improved from baseline. Confinement had a negative impact on the general health, diet, sleep and physical activity of this population (at risk of diabetes); however, weight and BMI decreased, and adherence to a Mediterranean diet improved.

## 1. Introduction

In March 2020, the WHO declared COVID-19 a global pandemic [1]. Following the increase in SARS-CoV-2 infections and observing the responses of many other countries, on March 15, 2020, a state of emergency was decreed in Spain [2]; the decree included general home confinement, which affected the majority of the population and sought to reduce the spread of the pandemic. In Spain, the confinement lasted until June, almost 100 days. It involved drastic changes in the day-to-day lives of the population; therefore, health habits were affected, as various studies have already reported [3,4,5,6]. There were significant impacts on the mental health of the population [7,8]; physical activity (PA) decreased [9]; sleep time and quality were altered [10]; and eating habits changed [11,12,13].

These impacts of general confinement applied at the population level have had important effects on society [14]. The effects that confinement may have on people with chronic diseases that are associated with lifestyle habits, for example, diabetes, or risk factors for developing chronic diseases are particularly relevant [15,16].

The high-risk intervention implemented through the ALAS programme includes individualised care and an intensive and structured group education workshop aimed at people with grade II overweight, obesity and/or a high risk of type 2 diabetes. The aim of the programme is to address and prevent these diseases by promoting changes in lifestyles, such as healthy eating, following a Mediterranean Diet and regular PA.

The programme began in 2011 and continues today. An effectiveness study was conducted with 1629 people who participated between 2016 and 2019 [17], concluding that (1) at the end of the intervention, 85% had lost weight, with 43% losing more than 5% of their baseline weight; (2) 22.3% of the people with obesity no longer presented obesity; (3) 35.1% of people classified as prediabetic according to the criteria of the American Diabetes Association became normoglycaemic; and (4) the effects on weight lasted at least 6 months after the end of the intervention.

Since September 2019, prior to confinement, 533 people have enrolled in the programme. Due to social isolation measures, the programme was interrupted. The participants who had started the intervention and the workshops were confined to their homes.

The purpose of this study is to describe the impact of confinement on the lifestyles of this sample of people with excess weight and/or a high risk of diabetes who had initiated an intervention programme to modify their health status.

## 2. Materials and Methods

### 2.1. Design

This was a descriptive study conducted in Madrid from May to June 2020, when the general confinement measures were de-escalated. A questionnaire was administered by telephone to people who were implementing the intervention programme. The interviews were conducted by health professionals affiliated with the programme and served simultaneously to maintain contact with the participants. Some data collected (weight, BMI, adherence to a Mediterranean diet) could be compared with baseline data collected previously for the programme. Therefore, a pre–post quasi-experimental design was possible.

### 2.2. Participants

The target population was the 553 people who had started the workshop in the ALAS programme between September 2019 and March 2020. The inclusion criteria to enter the programme were to be over 18 years of age and have one of the following: a BMI over 30 or BMI between 27 and 30 with an abdominal circumference risk (greater than 88 cm in women or 102 cm in men) or a FINDRISC (Finnish type 2 Diabetes Risk Score) greater than 14, this instrument having been validated for the Spanish context [18]. Participants accessed the programme through municipal health centres or were referred by municipal occupational health services (aimed at city council workers).

### 2.3. Description of the Programme

The ALAS intervention, based on the Diabetes Prevention Programme [19], consists of an intensive and structured intervention lasting 6 months that includes an individual intervention programme and a group education workshop [20] of 10 sessions (2 h each), over a period of 6 months, aimed at reducing weight, improving diet and increasing PA. The specific objectives of the programme include a 5% reduction in body weight, improvement in adherence to a Mediterranean diet, improvement in participation in PA and improvement in glycaemic status (for those with prediabetes).

### 2.4. Variables

The questionnaire, which combined ad hoc-designed impact measurements with validated tools, had different blocks:

#### 2.4.1. Sociodemographic and Participation data

Age, gender, country of birth, marital status, education level and employment status were collected. Information was also collected on housing and cohabitants during confinement: number of cohabitants, cohabitants under 14 years of age (yes/no), cohabitants older than 65 years of age or dependents (yes/no), size of residence (square metres) and access to the internet from home (Yes/No). With these variables, the density of inhabitants per household was calculated in square metres per inhabitant.

The following variable was collected for participation in the programme: number of sessions until interruption.

#### 2.4.2. Health Status and Lifestyle Data

General health status: General health status before and after confinement was assessed through a Likert-type scale with 5 response options: very good, good, fair, poor and very poor.

Health-related life habits: Diet before and after confinement was assessed using a Likert-type scale with 5 response options (from 1 (unhealthy) to 5 (very healthy)). Participants were asked about changes in 12 practices related to diet that may have occurred due to confinement (better/same/worse): quantity of food consumed; variety of foods; regularity of meal times; snacking between meals; consumption of fresh, packaged and processed foods, sweets, pastries, soft drinks and alcoholic beverages; menu planning; time dedicated to cooking; and financial ability to implement a healthy diet. A validated questionnaire on adherence to a Mediterranean diet (MEDAS 14) [21] is used as an assessment tool for the programme. The questionnaire has 14 items related to the weekly frequency of the consumption of certain products, such as vegetables, oil and legumes. Each item has a cut-off value based on recommendations, and the global scale differentiates among high, medium and low adherence to a Mediterranean diet. Regarding physical exercise, the number of days per week and the average time spent exercising before and after confinement were assessed. Sleep time (total, day and night) and sleep quality (reduced or maintained) during confinement were also investigated.

#### 2.4.3. Anthropometric Data

While baseline data were collected by programme professionals taking actual measurements, weight and height during confinement were self-reported, from which the BMI was calculated.

These data were paired to baseline data (weight, BMI and diet adherence) collected through the programme at the beginning of the intervention while maintaining anonymity. Variations in weight, BMI and diet adherence before and after confinement were calculated. In addition, the percentage of people who had achieved a 5% reduction in baseline weight was calculated; this variable is one of the main evaluation objectives of the ALAS programme.

### 2.5. Analysis

A descriptive analysis of all data was performed. The health and lifestyle variables were crossed, and chi-square tests and ANOVA were used to explore bivariate relationships. The factors related to the deterioration of general health or diet were adjusted using logistic regression models; the adjusted ORs are presented with the corresponding confidence intervals. The models were adjusted for all of the variables that had a significance of at least 90% and whose omission did not alter the parameters of the remaining variables by more than 5%.

For the pre–post analysis, weight, BMI, % obesity and adherence to a Mediterranean diet were compared using t-tests. Logistic regression analysis was performed to adjust the factors related to achieving a 5% reduction in baseline weight.

### 2.6. Ethical Aspects

The study complied with the principles of the Declaration of Helsinki of 2013. All participants gave their informed consent to answer the questionnaire and were informed that their collaboration was voluntary, anonymous and not conditional on their subsequent participation in the programme. The study was approved by and received ethical consent from the municipal health promotion service of Madrid Salud, which ensured anonymity throughout the process.

## 3. Results

### 3.1. Description of the Sample

A total of 387 (72.6%) of the 533 participants enrolled in the ALAS programme from September 2019 to February 2020 responded to the survey. There were no differences between the participants and non-participants in terms of age and sex, although the participants had a higher percentage of people with obesity at baseline (69.4 vs. 59.1) and therefore higher weight, waist circumference and baseline score of FINDRISK.

The mean age of the participants was 57.5 years (SD = 12.1; Rank = 18 − 86), and 77.9% of the sample were women. A total of 25.6% had not participated in any workshop sessions, 42% had participated in 1 to 5 sessions, and 32.4% had participated in 6 or more (complete intervention according to the ALAS programme protocol). The sociodemographic and housing characteristics of the sample are shown in Table 1, stratified by gender.

### 3.2. Impact on Self-Perceived Health

Before confinement, 73.9% of the participants claimed to have good or very good health, with 19.4% and 6.7% reporting fair and bad or very bad health, respectively; during confinement, these percentages changed to 53.2%, 33.9% and 12.9%, respectively. A total of 31.8% of the participants reported a worse perception of their health after confinement, with 63.1% maintaining their self-perception of health and 5.1% reporting an increase. The worsening of the self-perceived state of health after the onset of the pandemic was related to being affected by obesity, being single, separated or divorced, and living with dependents; in contrast, living with minors was associated with preventing a deterioration in health (Table 2). When these factors were adjusted in a logistic regression model that included age and self-perceived health status before the pandemic, the effect of living with minors as a protective factor against deterioration was maintained (OR = 0.16; 95% CI = 0.06−0.43), and obesity was maintained as a risk factor (OR = 2.41; 95% CI = 1.36−4.28).

### 3.3. Impact on Sleep Habits

A total of 28.3% of the participants reduced their total sleep time during confinement, 59.3% maintained it, and 12.4% increased it. Regarding sleep quality, 39.6% believed that it worsened during confinement; for 53.9%, sleep quality remained the same, and for 6.5%, it improved. Sleep time at night decreased for 35% of the people surveyed, was similar to that before confinement for 55.5% and increased for 9.4%. Simultaneously, sleep time during daylight hours increased for 15.4% of the people surveyed, remained the same for 75.7% and decreased for 8.9%.

The worsening of sleep quality was related to being a woman or being unemployed (*p* < 0.05). Sleeping less during the day was statistically significantly related to having children or living with older dependents. The worsening of general health and diet during confinement was also significantly related to a worse quantity and quality of sleep. Age was also a factor. Younger people had greater sleep disturbances and worse sleep time and quality than did older people: 51.1% of participants under 45 years of age perceived worse sleep quality during confinement, followed by those who were 45–54 years of age, 43%; between 55 and 64 years of age, 40.3%; and those over 65 years of age, 31.5%.

### 3.4. Impact on Physical Exercise

Before confinement, 8.1% of the participants did not perform any type of weekly physical exercise; after confinement, this percentage increased to 28.6%, while 61% decreased the time they dedicated to physical exercise, with 24.4% reporting an increase and 14.6% reporting no change. Participants were physically active, on average, for 5 h per week (range: 0–35; SD = 4.2) before confinement and 3.4 h on average after confinement (range: 0–35; SD = 4). Before confinement, 60.3% met the recommendation of 30 min of physical exercise daily; after confinement, this percentage decreased to 40.8%.

The impact of confinement on PA was related to previous participation in PA (Table 3). A reduction in PA was related to greater-than-average participation in PA previously. In contrast, an increase in PA was related to participating in less PA before confinement. The participants who reported an increase in PA did so for an average of 3.9 h per week (SD = 4.3), 173% (SD = 214.5) of their pre-pandemic PA time. Those who reduced their participation in PA did so by an average of 4.2 h per week (SD = 3.6), 72% of the pre-pandemic PA time (SD = 28.7). These variations were significant (t-test, *p* < 0.01). Among the people who increased their PA, 36.7% met the recommendation of 30 min per day before confinement, increasing to 84.4% after confinement; for those who reduced their PA, compliance decreased from 73.8% to 22.2%.

### 3.5. Impact on Diet

The average assessment of eating practised before confinement was 3.78 (SD = 0.89) on a scale of 1 to 5 (1: unhealthy; 5: very healthy). After confinement, the average was 3.47 (SD = 1.09), representing a significant difference (paired-samples Student’s t-test, *p* < 0.001). A total of 34% reported a worse diet (scored worse) after confinement, 53.4% maintained their diet without changes, and 12.2% improved their diet. The relationship between the characteristics of the sample and the impact of confinement on the self-perception of their diet is shown in Table 4, and the adjustment by logistic regression is shown in Table 5. The negative impact of confinement on diet, adjusted for the assessment of diet before confinement (those who had a better diet experienced a greater negative impact), was significantly related to being middle-aged (45–64 years), living in a residence with less than 25 m^2^ per cohabitant, living with elderly individuals or dependent people and being affected by obesity.

Regarding the 12 dietary practices that were assessed (Table 6), 89.4% of the participants had modified at least 1 during confinement (average of 4.3 practices changed). Modifying a practice (increasing or decreasing) was related to a self-perception of the deterioration of diet during the pandemic: 37.3% of those who modified their practices reported a worse diet, and among those who did not change dietary practices, 10.3% reported a worse diet.

People over 65 years of age changed their practices the least, those younger than 45 years reduced healthy practices the most, and those aged 45–54 years increased their unhealthy practices the most. All participants who lived with minors changed their eating practices: this change was significantly associated (*p* < 0.05) with increasing healthy practices and reducing unhealthy practices, with the exception of the financial ability to implement a healthy diet, which was significantly lower in households with minors than in the rest (19.6% vs. 7.9%).

Some healthy practices increased, such as spending more time in the kitchen and the consumption of fresh food, and others were reduced, such as the regularity of meal times or the variety of foods consumed. The same happened with less healthy practices: snacking between meals, the consumption of sweets and pastries, and the amount of food consumed increased, and the consumption of packaged and processed foods, soft drinks and alcoholic beverages was reduced.

### 3.6. Weight Variation, BMI and Adherence to a Mediterranean Diet

Complete pre- and post-confinement weight data were obtained for 84% of the sample (n = 325). The majority (64.3%) lost at least 1 kg of weight from the baseline measurement. A total of 16.9% maintained their weight, and for 18.8%, their weight increased. On average, the surveyed population lost 2.7 kg (SD = 4.9), i.e., 3.1% of their baseline weight (SD = 5.5). Among the people who gained weight, the average gain was 3.4 kg (SD = 3.1) or 4% of their baseline weight (SD = 4), and among those who lost weight, the average loss was 5.2 kg (SD = 4) or 6% of their baseline weight (SD = 4.1). Weight loss was related to having accessed the programme through municipal occupational health services and being initially classified as overweight (not presenting obesity). Weight loss was also inversely related to the consumption of processed foods: among the people who reduced this consumption, 89.9% lost weight; among those who maintained consumption, 60.7% lost weight; and among those who increased consumption, 55.3% lost weight.

The variations in weight, BMI and adherence to a Mediterranean diet are shown in Table 7. Of the 322 people with complete data, 68.6% were classified as affected by obesity, 25.8% were overweight and 5.6% were normal weight at baseline; after confinement, the classification percentages were 58.1%, 34.8% and 7.1%, respectively. A total of 14% had a lower classification level (from obesity to overweight or from overweight to normal weight), 84.5% remained in the same classification, and 1.6% increased. Among the people initially classified as affected by obesity, 16.7% became overweight.

The variables that were statistically significantly related (*p* < 0.05) to the decrease in BMI after confinement were access to the programme through the municipal occupational health service (27.3% reduced BMI, compared to 11.9% of those who accessed the programme through community health centres), overcrowding in the home (17.3% in homes with less than 25 m^2^/habitant, compared to 6.8% in those with more than 50 m^2^/habitant) and employment status (17.7% in workers and 15.5% in retirees vs. 2.6% in unemployed and none of those who were dedicated to home care). The variation in BMI was not significantly related to the number of sessions performed, to the abandonment of the intervention or to not having performed any sessions.

Of the 287 people with complete MEDAS-14 data, 17.5% had low adherence to a Mediterranean diet at baseline, 65.9% had medium adherence, and 16.5% had high adherence. After confinement and starting the ALAS intervention, 6.1% had low adherence, 63.3% had medium adherence, and 30.6% had high adherence. A total of 31.4% improved their adherence, 56.4% maintained it, and 12.2% lowered it. A total of 83.3% of participants with low adherence at baseline improved.

This variation in adherence was not significantly related to the characteristics of the sample or to the variables related to the implementation of the intervention (number of sessions, dropouts, not performing any session), although the people who had participated in at least one session improved adherence more than those who had not participated in any (34.1% vs. 21.9%; *p* = 0.064).

Thirty-two percent (104) of the sample with complete weight data lost at least 5% of their initial weight, one of the main objectives of the ALAS programme. The adjusted factors related to this objective (Table 8) were accessing the programme through municipal occupational health services, having high adherence to a Mediterranean diet, having improved menu planning, being of non-Spanish origin and living in households with 80 to 100 m^2^. Thirty-six percent of the people who completed three or more sessions of the ALAS workshop met the goal of a 5% or more reduction in their baseline weight, with 25.6% of those who did not participate in any sessions achieving the same goal; the difference was not statistically significant (*p* = 0.401 in the chi-square test).

## 4. Discussion

The results of this study indicate the important impact that general confinement has had on the lifestyles of people at high risk of having diabetes who have begun an intensive health promotion intervention. Although the majority of people maintained their self-perception of health, approximately one-third reported worse sleep and diet quality and decreased PA. Changes in feeding practices varied and had positive and negative effects. In the sample studied, there was a significant reduction in weight and therefore in BMI, as well as an improvement in adherence to a Mediterranean diet.

Although 63.1% of the participants stated that there were no changes in their general self-perception of health during confinement, 31.8% reported that it was worse. This negative impact of confinement on the self-perception of health in general [22,23] and mental health specifically [24] has been noted in various studies conducted in recent years. There are factors, such as the context in which confinement took place [22] or the existence of chronic diseases or previous mental health problems [25], that have been related to this negative impact; however, they were not measured in our study. For other factors that have been shown to have a relationship, such as gender, age and social position [26], and that were collected here, the relationships were not maintained after adjustment. For our participants, being affected by obesity most conditioned the negative impact. While the relationship between obesity and poor health perception is known [27], there is little evidence about the relationship between obesity and the impact of confinement, although the media and scientific publications have already highlighted people with obesity as a potentially vulnerable population in the face of the imminence of the pandemic [28].

When adjusted for other factors, living with children (regardless of gender) emerged as a protective factor against the worsening of health or diet perceptions. This is an unexpected result; it is possible that confinement offered, among many difficulties, opportunities to improve care and dedicate more time to cohabitant minors. In fact, people with children were among those who had to make the most changes in their routines during confinement [29,30]. Other studies of confinement indicate that living with minors is related to a negative impact on diet [31]; however, the previous literature is divided as to whether it worsens or improves the household diet [32,33].

Our data indicate that the majority of participants maintained their usual sleep time, while 28% reduced their sleep time and 12% increased it. However, up to 40% reported worse sleep quality. A French study [34] and an Australian study [35] also found increases in the incidence of sleep problems compared to relevant evidence before confinement. Others [36,37] have also observed this increase, describing associated factors such as overexposure to blue light due to the increase in the use of devices and in teleworking.

Our results indicate a general decrease in the time dedicated to PA during confinement. This result is consistent with various studies in the general population conducted for the same period in Spain [38,39] and in approximately 20 studies in Italy reviewed by Zaccagni et al. [40]. The results also coincide with a Singaporean investigation based on data recorded automatically and continuously by bracelets [41]. In contrast, a few studies in European countries obtained contradictory results that indicate an increase in declared PA during confinement [42,43,44]. Consistent with our results, low compliance with the minimum recommended PA guidelines was also observed among the general population in Spain [39] and the United Kingdom [42].

Our data indicate that the decrease in PA was not homogeneous for all profiles: those who reported a higher level of PA before confinement experienced a strong decrease in compliance with the recommendation of 30 min of daily exercise, and for those who were less active pre-confinement, compliance with this standard significantly increased. This finding contrasts with the results of a study from Singapore [41]: i.e., people who decreased their PA the most were the most inactive before confinement, while those who were the most active pre-confinement maintained a profile with greater PA, despite a reduction.

Castañeda-Babarro [38] observed a similar reduction in PA that was more intense among originally more active individuals, a finding that could be explained by the forced closure of sports facilities during confinement and the lack of sports equipment at home. Additionally, among the most inactive people before confinement, moderate PA at home increased [43]. For the most inactive population, confinement represented an opportunity to dedicate time to PA, while those who were more active may have been limited by the inaccessibility of the spaces where they generally practised PA (gyms, swimming pools, parks, etc.).

Although half of the participants believed that there was no impact, one in three participants perceived that their diet had worsened with confinement. Several studies agree that diets were mostly maintained [5,43,45,46]. Some studies reported changes in eating habits; however, the direction of those changes is not clear [3,13]. A study by López-Moreno et al. [47] of the general population in Spain showed that 54% changed habits, with 38.4% improving and 16% worsening. Additionally, Net-Santé in France [31] indicated that 56.2% of participants changed their eating practices. As in our results, other studies agree that perceiving a deterioration in one’s diet is related to age [48] and BMI [49]; however, these studies do not relate this perception to the conditions of the home or to living with elderly dependents.

A study conducted in various European countries by Skotnicka et al. [50] concluded that people with worse eating habits before confinement experienced a more negative impact on their diets. In contrast, in our study, the results indicate the opposite. Similar results were observed in a follow-up of US and UK cohorts [4].

Among the variability in changes in eating habits, evidence from European studies supports the results obtained in our study, i.e., increased time spent cooking [3,31,43,47,48]; the increased consumption of fresh products, fruits and vegetables [3,12,31,39,43,48]; and the increased consumption of snacks and sweets [3,12,31,47,48,49,50,51]. In our study, one-fifth of the participants reduced their consumption of ultra-processed foods, a finding that is consistent with some studies [43] but that also differs from other studies that warned of an increase [3,47,52]. In our study, 18.1% of the participants increased their consumption of alcohol, and 10.5% reduced their consumption; therefore, the impact is not clear. In other European studies, high variability is noted in the impact on alcohol consumption [3], with results that indicate that it increases [47,48,49,50,51] and others that indicate that it decreases [12,39,43,53].

These changes in habits seem to have produced slight improvements in adherence to a Mediterranean diet [50,53], which is considered heart-healthy. In our study, a significant increase in this adherence stands out (31.4%); however, the majority (56.4%) did not report significant changes.

These impacts on changes in habits (eating and PA) produced weight changes in people who lived in confinement; however, these include increases and decreases. Therefore, there is substantial variability [11,31,47,48], and at the population level, there does not seem to be a significant effect [54,55]. A longitudinal study in the United Kingdom reported that there was an increase in average weight at the beginning of confinement but that weight later decreased, resulting in no impact of confinement on weight [54].

There seems to be some consensus that the people who gained the most weight were those who were previously affected by obesity or overweight [3,11,54,56]. This contrasts with the results of our sample: 68.6% were initially affected by obesity, and the majority (64.3%) lost at least 1 kg of weight and therefore reduced their BMI.

A total of 32% of the sample achieved a reduction of 5% in their baseline weight, one of the main objectives of the ALAS programme. Although the design of this study did not allow the establishment of causal relationships, several hypotheses can be proposed to explain how there was a significant reduction in weight in this sample, while most studies in Spain and nearby countries indicate that, at the population level, there was no significant impact. The participants had decided to enrol in a health education programme to modify their lifestyle; therefore, they were people in a phase of preparation for action according to the transtheoretical model [57,58] and therefore more likely to make these changes. In addition, for this population, the ALAS programme serves as a motivator and supports the implementation of change. In fact, the 32% efficacy in the 5% reduction in baseline weight is similar to the 34% established in previous evaluations of the programme [17] for participants who did not complete the intervention. That is, the simple fact of having access to the intervention (without completing it) may promote change. For this study, intervention follow-up (number of sessions performed) did not have a significant effect. By entering the programme (an inclusion criterion in this study), participants meet for an individual session where they are weighed, measured, given questionnaires on lifestyle habits and provided information and brief advice to motivate change. This intervention and brief advice by themselves have already demonstrated some efficacy, although slight in magnitude, in promoting change [58,59,60]. Thus, although confinement seems to impact the weight of the population in a variable direction that results in modest changes at the population level, other factors were also at play in our study, such as the selection of a sample motivated to change and the fact that they were already enrolled in a health promotion programme. These last factors seem to have counteracted the effects of confinement described in other studies of the general population, particularly among people with obesity and overweight people.

Although the ALAS programme is carried out in municipal health centres, participants can either be recruited directly in these centres or start after a referral from the labour services of the city council. Those who enrol in the programme through the second path are mainly municipal officials, meaning they are civil servants, a socio-economic profile that is typically subjected to more favourable social determinants than the general population captured in universal access services. This may explain why the pre–post results of the intervention are better in the group of people who accessed the programme through an occupational health referral, as happened in the evaluation of the ALAS programme itself [17].

The study had a very high participation rate (72.6%), which is very valuable, given the difficult moment in which the questionnaire was administered. This success is attributed to the following: unlike most studies conducted during confinement, instead of opting for an online questionnaire, a telephone interview was conducted by professionals from their municipal health centres. Using this approach, barriers to the accessibility of online questionnaires were overcome; therefore, our sample has a higher proportion of elderly people and people with low levels of education than do other studies conducted in Spain in the same period [23,26,29,39,47,50,53]. Additionally, our sample is of particular interest for evaluating the impact of confinement because the participants were selected precisely because they are people with obesity and/or have a high risk of developing diabetes in the next 10 years if they do not modify their lifestyles. As in most studies carried out during confinement and referenced in this work, weight during confinement, as well as some of the impacts of confinement, was self-reported. This can potentially produce some biases; these should be similar to those that occur in the general population in other studies, and therefore, they should be comparable. There were statistically significant differences in the baseline weight of the participants compared to the non-participants, and a potential participation bias was checked. In this case, the results go against the hypothesis, because those who did not participate were precisely the ones who weighed less, so we understand that there was not a significant bias. Additionally, complete pre–post data on weight were not available for 62 participants (16% of the sample). Half of these cases were due to the fact that the baseline information could not be collected due to the start of the pandemic. The other half did not know their weight during confinement, as they may not have had the instruments to measure it. We believe that these data losses are not important enough to generate a bias in the study.

## 5. Conclusions

A significant percentage of people at risk of developing chronic diseases who enrolled in the ALAS health promotion programme between October 2019 and February 2020 perceived that the confinement experienced between March and June 2020 had a negative impact on their overall health, diet, sleep habits and PA. Interestingly, when compared with baseline data, weight and BMI decreased, and adherence to a Mediterranean diet improved. This positive effect on health could be explained by the participants being prepared to change and finding motivation and support through the ALAS programme. These results suggest that the development of public health promotion programmes with high-risk populations not only has a potential direct effect on their participants but can also protect them from the effects of unforeseen adverse situations threatening the stability of their health-related lifestyles, such as general confinement in this case. This could orient policymakers to continue promoting programmes targeting specific vulnerabilities and striving to sustain these interventions even in times of emergency. Our discussion also suggests that the satisfactory evolution of the participants during confinement might be related to the fact that the participants seem to be in an adequate phase of preparation for changes in their lifestyles. This leads to the recommendation that health promotion programmes adapt their interventions at the stage of change in the participants, i.e., raise motivation among those who do not present that attitude before prescribing specific routine transformations among those who are willing to introduce changes in their lifestyles. In any case, more research is needed to strengthen these ideas.

## Figures and Tables

**Table 1:** Sociodemographic and housing characteristics of the sample by gender.

	Total N (% Column)	Men N (% Column)	Women N (% Column)	*p* *
Sample	387	85	299	-
Sociodemographic variables				
Age				
≤44 years	52 (13.7)	13 (15.3)	39 (13.4)	0.932
45–54 years	81 (21.4)	18 (21.2)	63 (21.6)	
55–64 years	134 (35.4)	31 (36.5)	102 (39.4)	
≥65 years	112 (29.6)	23 (27.1)	88 (30.1)	
Origin				
Spain	349 (90.4)	80 (94.1)	267 (89.3)	0.498
Latin America	32 (8.3)	5 (5.9)	27 (9)	
Other	5 (1.3)	-	5 (1.7)	
Marital status				
Married or with a partner	219 (57.6)	61 (74.4)	157 (53.8)	0.001
Single	74 (19.5)	16 (19.5)	58 (19.9)	
Separated or divorced	43 (11.3)	2 (2.4)	40 (13.7)	
Widowed	40 (10.5)	3 (3.7)	37 (12.7)	
Complete studies				
Primary or lower	128 (34.6)	24 (29.3)	102 (35.7)	0.492
Secondary	45 (12.2)	13 (15.9)	32 (11.2)	
Baccalaureate or VE	92 (24.9)	19 (23.2)	73 (25.5)	
University	105 (28.4)	26 (31.7)	79 (27.6)	
Employment situation				
Working	153 (41.2)	38 (46.9)	115 (39.9)	0.038
Unemployed	47 (12.7)	5 (6.2)	42 (14.6)	
Retired/pension	146 (39.4)	37 (45.7)	107 (37.2)	
Home care	23 (6.2)	1 (1.2)	22 (7.6)	
Student	2 (0.5)	-	2 (0.7)	
Housing variables				
Number of cohabitants				
One	81 (21.8)	9 (11)	71 (24.7)	0.038
Two	151 (40.7)	41 (50)	109 (38)	
Three	73 (19.7)	15 (18.3)	58 (20.2)	
More than three	66 (17.8)	17 (20.7)	49 (17.1)	
Household size (m^2^)				
≤60	73 (20.6)	17 (22.1)	55 (20)	0.014
61–80	136 (38.4)	26 (33.8)	109 (39.6)	
81–100	84 (23.7)	12 (15.6)	72 (26.2)	
>100	61 (17.2)	22 (28.6)	39 (14.2)	
Density of household inhabitants (m^2^x cohabitant)				
≤25	91 (25.8)	19 (25)	72 (26.2)	0.972
25.5–37.5	95 (26.9)	22 (28.9)	72 (26.2)	
38–50	66 (18.7)	14 (18.4)	52 (18.9)	
>50	101 (28.6)	21 (27.6)	79 (28.7)	
Cohabitants in charge				
Children	52 (14)	12 (14.5)	39 (13.6)	0.849
Seniors/dependents	45 (12.2)	8 (9.8)	37 (13)	0.432
With internet access	319 (86.7)	73 (88)	244 (86.2)	0.684

* *p* value for the chi-square test of the relationship between the variable and gender, where <0.05 is considered a significant relationship.

**Table 2:** Factors related to the deterioration of self-perceived health during confinement.

	Worse Health Status N (% row)	Not Worse Health Status N (% row)	*p* *
Sample	118 (31.8)	253 (68.2)	
Age			
≤44 years	17 (37.8)	28 (62.2)	0.117
45–54 years	32 (41)	46 (59)	
55–64 years	41 (31.5)	89 (68.5)	
≥65 years	28 (25.2)	83 (74.8)	
Gender			
Men	27 (32.5)	56 (67.5)	0.903
Women	91 (31.8)	195 (68.2)	
Pre-confinement BMI classification			
Normal weight	4 (22.2)	14 (77.8)	0.004
Overweight	19 (22.1)	67 (77.9)	
Obesity	94 (40.9)	136 (59.1)	
Origin			
Spain	104 (30.9)	233 (69.1)	0.218
Other	14 (41.2)	20 (58.8)	
Marital status			
Married or with a partner	60 (28.2)	153 (71.8)	0.020
Single	18 (41.9)	25 (58.1)	
Separated or divorced	30 (42.9)	40 (57.1)	
Widowed	8 (20)	32 (80)	
Complete studies			
Primary or lower	31 (25.2)	92 (74.8)	0.129
Secondary	13 (29.5)	31 (70.5)	
Baccalaureate or VE	34 (38.2)	55 (61.8)	
University	39 (37.5)	65 (62.5)	
Employment situation			
Working	52 (35.1)	96 (64.9)	0.559
Unemployed	15 (34.1)	29 (65.9)	
Retired/pension	43 (29.5)	103 (70.5)	
Home care	5 (22.7)	17 (77.3)	
Access through occupational health			
Yes	14 (29.2)	34 (70.8)	0.674
No	104 (32.2)	219 (67.8)	
Number of cohabitants			
One	30 (37)	51 (63)	0.219
More than one	86 (29.9)	202 (70.1)	
Household size (m^2^)			
≤60	25 (34.7)	47 (65.3)	0.325
61–80	38 (27.9)	98 (72.1)	
81–100	33 (39.3)	51 (60.7)	
>100	18 (29.5)	43 (70.5)	
Density of household inhabitants (m^2^x cohabitant)			
≤25	34 (37.8)	56 (62.2)	0.259
25.5–37.5	31 (32.6)	64 (67.4)	
38–50	15 (22.7)	51 (77.3)	
>50	33 (32.7)	68 (67.3)	
Cohabiting minors			
Yes	5 (9.8)	46 (90.2)	0.000
No	112 (35.1)	207 (64.9)	
Cohabiting elderly individuals/dependents			
Yes	21 (46.7)	24 (53.3)	0.022
No	96 (29.7)	227 (70.3)	
Internet access			
Yes	107 (33.6)	211 (66.4)	0.118
No	11 (22.4)	38 (77.6)	

* *p* value for the chi-square test of the relationship between the variable and worsening health status, where <.05 is considered a significant relationship.

**Table 3:** Relationship of the impact of confinement on physical activity based on previous activity.

	All	Increase PA	Maintain PA	Reduce PA	*p*
(% row)	100	24.4	14.6	61.0	-
Weekly PA time PRE (hours x week)	5.0	2.9	3.9	6.1	<0.01 *
Weekly PA time POST (hours x week)	3.4	6.8	3.9	1.8	<0.01 *
Variation in weekly PA time PRE–POST (hours x week)	1.6	3.9	-	−4.2	<0.01 *
Mean % of PA time variation (% of PRE time)	−8.7	173	-	−72.6	<0.01 *

PA = physical activity. * ANOVA of the time dedicated to PA according to impact (increase/maintain/decrease).

**Table 4:** Relationship between factors and the impact of confinement on diet.

	Worse Diet N (% Row)	Maintained Diet N (% Row)	Improved Diet N (% Row)	*p* *
Sample	127 (34.4)	197 (53.4)	45 (12.2)	
Age				
≤44 years	16 (35.6)	20 (44.4)	9 (20)	0.000
45–54 years	35 (44.9)	31 (39.7)	12 (15.4)	
55–64 years	54 (41.9)	63 (48.8)	12 (9.3)	
≥65 years	20 (18.2)	79 (71.8)	11 (10)	
Gender				
Men	27 (32.9)	45 (54.9)	10 (12.2)	0.929
Women	100 (35.1)	150 (52.6)	35 (12.3)	
Pre-confinement BMI classification				
Normal weight	4 (22.2)	12 (66.7)	2 (11.1)	0.023
Overweight	19 (22)	55 (64)	12 (14)	
Obesity	94 (40.9)	110 (47.8)	26 (11.3)	
Origin				
Spain	109 (32.6)	181 (54.2)	44 (13.2)	0.040
Other	18 (51.4)	16 (45.7)	1 (2.9)	
Marital status				
Married or with a partner	75 (35.7)	115 (54.8)	20 (9.5)	0.012
Single	19 (44.2)	20 (46.5)	4 (9.3)	
Separated or divorced	25 (35.2)	31 (43.7)	15 (21.1)	
Widowed	6 (15)	29 (72.5)	5 (12.5)	
Complete studies				
Primary or lower	34 (27.6)	74 (60.2)	15 (12.2)	0.012
Secondary	17 (37.8)	27 (60)	1 (2.2)	
Baccalaureate or VE	35 (39.8)	46 (52.3)	7 (8)	
University	37 (35.9)	45 (43.7)	21 (20.4)	
Employment situation				
Working	62 (41.9)	65 (43.9)	21 (14.3)	0.006
Unemployed	21 (46.7)	21 (46.7)	3 (6.7)	
Retired/pension	35 (24.3)	92 (63.9)	17 (11.8)	
Home care	6 (28.6)	14 (66.7)	1 (4.8)	
Access through occupational health				
Yes	20 (41.7)	22 (45.8)	6 (12.5)	0.488
No	107 (33.3)	175 (54.5)	39 (12.1)	
Number of cohabitants				
One	21 (37.1)	147 (51.4)	33 (11.5)	0.170
More than one	106 (25.9)	48 (59.3)	12 (14.8)	
Household size (m^2^)				
≤60	22 (30.1)	44 (60.3)	7 (9.6)	0.653
61–80	44 (32.6)	72 (53.3)	19 (14.1)	
81–100	34 (41)	38 (45.8)	11 (13.3)	
>100	19 (31.7)	34 (56.7)	7 (11.7)	
Density of household inhabitants (m^2^x cohabitant)				
≤25	48 (52.7)	37 (40.7)	6 (6.6)	0.002
25.5–37.5	28 (29.5)	53 (55.8)	14 (14.7)	
38–50	18 (28.1)	36 (56.3)	10 (15.6)	
>50	25 (25)	61 (61)	14 (14)	
Cohabiting minors				
Yes	107 (33.9)	173 (54.7)	36 (11.4)	0.290
No	20 (38.5))	23 (44.2)	9 (17.3)	
Elderly individuals or cohabiting dependents				
Yes	102 (31.7)	179 (55.6)	41 (12.7)	0.016
No	24 (53.3)	17 (37.8)	4 (8.9)	
Internet access				
Yes	9 (18.4)	34 (69.4)	6 (12.2)	0.026
No	118 (37.3)	159 (50.3)	39 (12.3)	

* *p* value for the chi-square test of the relationship between the variable and worsening health status, where <0.05 is considered a significant relationship.

**Table 5:** Adjusted effects of variables related to post-confinement diet deterioration.

	Adjusted OR	95% CI
Cohabiting with elderly individuals or dependents		
No	Ref	-
Yes	2.55	1.22–5.32
Age		
≤44 years	1.47	0.58–3.73
45–54 years	3.16	1.42–7.02
55–64 years	2.51	1.27–4.96
≥65 years	Ref	-
Density of household inhabitants		
≤25 m^2^x cohabitant	2.70	1.26–5.78
25.5–37.5 m^2^x cohabitant	1.10	0.53–2.31
38–50 m^2^x cohabitant	1.68	0.74–3.78
>50 m^2^x cohabitant	Ref	-
Pre-confinement BMI classification		
Normal/overweight	Ref	-
Obesity	2.24	1.19–4.21
Assessment of diet BEFORE confinement (scale 1–5)	1.54	1.12–2.12

**Table 6:** Change in dietary practices during confinement.

	Change in Practices during Confinement		
	Less N (% Row)	Same N (% Row)	Greater N (% Row)
Healthy practices			
Time spent cooking	24 (6.5)	217 (58.6)	129 (34.9)
Planning menus	61 (16.5)	244 (65.9)	65 (17.6)
Consumption of fresh food	43 (11.6)	267 (72.2)	60 (16.2)
Variety of foods	64 (17.3)	257 (69.5)	49 (13.2)
Regularity of schedules	66 (17.8)	270 (73.0)	34 (9.2)
Financial ability to have a healthy diet	35 (9.5)	325 (87.8)	10 (2.7)
Unhealthy practices			
Unhealthy snacking between meals	55 (14.9)	150 (40.7)	164 (44.4)
Consumption of sweets and pastries	64 (17.3)	167 (45.3)	138 (38.4)
Amount of food consumed	64 (17.3)	173 (46.6)	134 (36.1)
Consumption of soft drinks	72 (19.5)	251 (67.5)	47 (12.7)
Consumption of packaged/processed foods	76 (20.5)	252 (68.1)	42 (11.4)
Consumption of alcoholic beverages	67 (18.1)	264 (71.4)	39 (10.5)

**Table 7:** Variations in weight, BMI and adherence to a Mediterranean diet.

	N	Pre	Post	Variation	*p* *
Weight (average kg)	325	86.9	84.2	2.7	<0.01
BMI	322	32.97	31.93	1.03	<0.01
% With obesity	322	68.6	58.1	10.6	<0.01
% High adherence to a Mediterranean diet	287	18.5	29.6	11.1	<0.01

* *p* value for the t-test of the measurement of the paired variable before and after confinement.

**Table 8:** Adjusted effect of the factors related to a 5% reduction in baseline weight.

	Adjusted OR	95% CI
Access through occupational health		
No	Ref	-
Yes	4.93	2.09–11.61
Adherence to a Mediterranean diet		
Medium or low	Ref	
High	2.76	1.48–5.13
Planning menus		
Less than or equal	Ref	
Major	2.53	1.22–5.25
Origin		
Spain	Ref	
Other	3.28	1.25–8.64
Household size		
≤ 60 m^2^	2.15	0.75–6.13
61–80 m^2^	1.47	0.57–3.77
81–100 m^2^	3.13	1.19–8.27
> 100 m^2^	Ref	
Density of household inhabitants		
≤ 25 m^2^x cohabitant	1.17	0.50–2.69
25.5–37.5 m^2^x cohabitant	0.63	0.28–1.41
38–50 m^2^x cohabitant	2.15	0.94–4.94
>50 m^2^x cohabitant	Ref	

## Data Availability

The datasets used and/or analysed during the current study are available from the corresponding author on reasonable request.
